# Development and In Vitro-In Vivo Evaluation of a Novel Sustained-Release Loxoprofen Pellet with Double Coating Layer

**DOI:** 10.3390/pharmaceutics11060260

**Published:** 2019-06-05

**Authors:** Dongwei Wan, Min Zhao, Jingjing Zhang, Libiao Luan

**Affiliations:** 1 College of Pharmacy, China Pharmaceutical University, No. 639 Longmian Road, Nanjing 211100, China; 710176323@163.com (D.W.); 19850856528@163.com (M.Z.); 15651915012@163.com (J.Z.); 2 College of Pharmacy, China Pharmaceutical University, Xuanwumen Campus, No. 24 Tongjiaxiang, Nanjing 210009, China

**Keywords:** sustained release pellets, double coating layer, loxoprofen, citric acid, pharmacokinetic studies

## Abstract

This study aimed to develop a novel sustained release pellet of loxoprofen sodium (LXP) by coating a dissolution-rate controlling sub-layer containing hydroxypropyl methyl cellulose (HPMC) and citric acid, and a second diffusion-rate controlling layer containing aqueous dispersion of ethyl cellulose (ADEC) on the surface of a LXP conventional pellet, and to compare its performance in vivo with an immediate release tablet (Loxinon^®^). A three-level, three-factor Box-Behnken design and the response surface model (RSM) were used to investigate and optimize the effects of the citric acid content in the sub-layer, the sub-layer coating level, and the outer ADEC coating level on the in vitro release profiles of LXP sustained release pellets. The pharmacokinetic studies of the optimal sustained release pellets were performed in fasted beagle dogs using an immediate release tablet as a reference. The results illustrated that both the citric acid (CA) and ADEC as the dissolution- and diffusion-rate controlling materials significantly decreased the drug release rate. The optimal formulation showed a pH-independent drug release in media at pH above 4.5 and a slightly slow release in acid medium. The pharmacokinetic studies revealed that a more stable and prolonged plasma drug concentration profile of the optimal pellets was achieved, with a relative bioavaibility of 87.16% compared with the conventional tablets. This article provided a novel concept of two-step control of the release rate of LXP, which showed a sustained release both in vitro and in vivo.

## 1. Introduction

Pellets, as multiple unit preparations, offer a lot of clinical benefits compared with single unit dosage forms, such as reduced intra- and inter-subject variability on drug plasma, decreased local irritations, less dose dumping risk, and stable plasma concentrations [1,2]. To prepare sustained release pellets, film coating is an ideal method. With the development of aqueous-based dispersion systems, film coating technologies have shifted from organic-based polymeric solutions to aqueous-based polymeric dispersion systems [3]. As one of the aqueous ethyl cellulose dispersions, Surelease^®^ (ADEC) could be used alone or combined with other polymers to obtain satisfactory release profiles [4,5,6,7]. Additionally, most of these release profiles showed a diffusion-controlled release mechanism, which meant a predictable release pattern could be achieved by altering the ADEC coating weight gain [8,9].

Loxoprofen sodium (LXP), as a 2-phenylpropinate non-steroidal anti-inflammatory drug (NSAD), was first introduced by Sankyo Company in Japan. It has been widely used for the treatment of osteoarthritis, scapulohumeral periarthritis, rheumatoid arthritis, arthritis, toothache, and post-operation pain [10]. As a pro-drug, LXP is converted to its active metabolite (trans-OH LXP) in vivo to inhibit the activity of cycloosygenase (COX), which mediates the production of inflammatory prostaglandins [11]. Due to the short elimination half-life of approximately 65 min [12], the commercial tablet of LXP has to be administrated three times a day to maintain the therapeutic concentration in plasma, which might cause high risks of gastrointestinal (GI) lesions and systemic side effects [13,14]. Several studies have been reported on the preparation of LXP sustained release dosage forms [15,16,17]. However, due to its high solubility, most of the preparations, especially for the matrix-based formulations, showed a burst release (drug release >30%) during the first 2 h [18], which could cause unexpected GI mucosal injury for patients. Therefore, a sustained release dosage form with decreased initial release would be necessary.

As a weakly acidic drug, loxoprofen shows good solubility at high pH, while poor solubility at low pH. Several strategies have been developed to prepare sustained release formulations of the pH-sensitive drugs [19,20,21,22]. Among them, incorporation of pH-modifiers into the preparation was a common approach in matrix or coating systems. These pH modifiers could significantly modify the micro-environmental pH (pH_M_) inside the systems, and result in a decrease or increase of the drug solubility, leading to a modified drug dissolution rate [22,23,24]. In addition, their extent and duration played an important role on the drug release rate [25]. Approaches like using coated pH-modifier as the starting core [23], blending pH-modifier with drugs into the core with a subsequent coating [26], or incorporating pH-modifiers into the matrix formulations [20], have been proposed and studied to achieve the sustained release of pH-sensitive drugs. However, for the maintenance of an appropriate pH_M_ inside the dosage forms, more than 20% pH-modifiers in the preparations were often needed [20,23,26], which might cause undesired GI irritations, especially for patients with GI ulcers. In order to reduce the usage of pH-modifiers and maintain an appropriate pH_M_ in the dosage form, citric acid (CA) as the pH-modifier was first proposed to be incorporated into the dissolution-rate controlling layer to decrease the dissolution rate of LXP.

Drug delivery systems (DDS), based on their system design or rate-controlling mechanism, can be divided into models such as dissolution, diffusion, erosion, osmosis, and swelling [27]. As for the film coating systems, the diffusion or osmosis mechanisms were often applied to elucidate the drug release profiles [5,28,29,30], while the influence of drug dissolution rate was often omitted or just attributed to the drug diffusion rate [27]. In the most common cases, only one of these mechanisms was applied to control the drug release rate in DDS, except for the bio-erodible or hydrogel matrix systems, where the drug release rate was controlled by two or three of these release mechanisms [31,32]. Although theoretical approaches regarding the dissolution-diffusion mechanism have been extensively reported [33,34], a combination of the dissolution and diffusion release mechanisms as a rate-controlling strategy was seldom reported.

**Figure 1:**
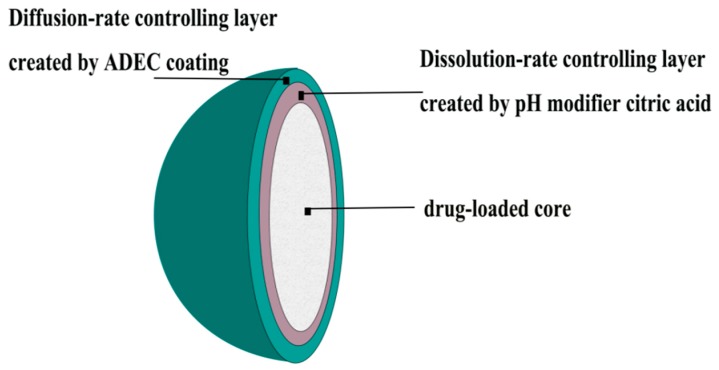
Schematic diagram of the sustained release pellets.

In this study, a novel concept of two-step control of the drug release rate is proposed. A schematic diagram of this hypothesis is illustrated in Figure 1. In this system, the first-step control was to reduce the dissolution rate of LXP by creating a sub-coating layer containing pH-modifier CA, while the second-step control was to decrease the diffusion rate of LXP by creating a non-soluble polymeric film. Furthermore, a three-level, three-factor Box-Behnken experiments design was conducted to optimize and evaluate the effects of different parameters on the drug release. Additionally, the pharmacokinetic studies of the optimal formulation were performed in fasted beagles to compare its in vivo performance with the conventional tablet.

## 2. Materials and Methods 

### 2.1. Materials

LXP dihydrate was purchased from Fujian Hui Tian biological Pharmaceutical Co., Ltd. (99.1% purity, China). Loxonin^®^ tablets were purchased from Daiichi Sankyo Co., Ltd. (Tokyo, Japan). Ketoprofen was purchased from Sigma (St. Louis, MO, USA). Microcrystalline cellulose SH-101 and corn starch were supplied by Sunhere Pharmaceutical Co., Ltd. (Huainan, China). Hydroxypropylmethyl cellulose (Methocel E5LV) and Surelease^®^ E-7-7050 (aqueous ethyl cellulose dispersion) were supplied by Colorcon Ltd. (Dartford, Kent, UK). Hard gelatin capsules were from Suzhou Capsugel Ltd. (Suzhou, China). Other chemicals were of reagent grade or higher grades.

### 2.2. HPLC-Assay for LXP and CA Contents

HPLC methods were used for the determination of LXP and CA. Chromatograph was carried out on Shimadzu LC-2030 (Shimadzu, Japan), equipped with an autosampler and an SPD-20A UV detector. The mobile phase used for the determination of LXP consisted of a mixture of methanol-water-acetic acid-triethylamine (600:400:1:1, *v/v*). Separation was achieved by applying an Inertsil C18 column (5 μm, 4.6 × 150 mm, Shimadzu, Japan), and the chromatogram was recorded at 223 nm. The limit of determination (LOD) and limit of quantitation (LOQ) for LXP were 0.03 and 0.1 μg/mL respectively. The mobile phase performed for the determination of CA was carried out with one part isopropanol and 999 parts 0.018 M phosphate buffer, adjusted to pH 2.5 with phosphoric acid. An Inertsil C18 column (5 μm, 4.6 × 150 mm, Shimadzu, Japan) was employed to separate the CA content. The flow rate was 1 mL/min, and the column temperature was controlled at 40 °C with the UV detection at 210 nm. The LOD and LOQ for CA were 0.2 and 0.6 μg/mL respectively. In addition, the calibration curve over the concentration range of 0.6–60 µg/mL had a regression coefficient of 0.9996.

### 2.3. Solubility/pH Profiles of LXP

Solubilities of LXP in different pH media were determined by adding excess of LXP to different buffer solutions: hydrochloric acid solutions (pH 1.2, 2.2), phosphate buffers (pH 3.0, 3.5, 4.0, 4.5 and 6.8). After vibrated at 25 °C for 24 h in a constant-temperature shaker (SHZ-82, Guohua Co., Ltd., Changzhou, China), 2 mL of the saturated solution was filtered through a membrane filter, and was diluted to avoid crystallization. Sample was determined by HPLC method.

### 2.4. Preparation of Drug-Loaded Pellets

The core consisted of loxoprofen sodium (37.1%, *w/w*), microcrystalline cellulose (41.9%, *w/w*), corn starch (19.9%, *w/w*), and talc (1.1%, *w/w*). Briefly, the mixture was blended in an ERWEKA mixer (Type AR YB5, Heusenstamm, Germany) at a speed of 40 rpm for 30 min. Then it was kneaded with the ethanol water solution (30%, *v/v*) in a laboratory kneader (Type LK5, Heusenstamm, Germany) for 10 min. The obtained moist mass was extruded at a speed of 500 rpm through a stainless steel barrel with a die of 0.8 mm diameter. Then, 300 g of the extrudates were processed in a 23 cm radial cut plate of spheronizer (JBZ-300, Liaoning New Drug Research Institute, China) at 1000 rpm for 10 min. The obtained pellets were collected on a Teflon tray and were dried in a hot oven at 60 °C for 24 h to remove the residual water and ethanol. Pellets with fraction size between 800 and 1250 µm were used for the following procedure.

### 2.5. Preparation of Sustained-Release Pellets

#### 2.5.1. Preparation of the Dissolution-Rate Controlling Layer 

Methocel E5 LV was added to purified water to achieve a hydroxypropylmethyl cellulose concentration of 4% (*w/w*). Then the solution was mixed with critic acid, with a concentration range from 0% to 6% (*w/w*), and was plasticized with PEG 6000 (0.5%, *w/w*) for 40 min [9]. Then, talc was added to the polymer mixture at a concentration of 2.5% (*w/w*). The aqueous suspension was stirred during the coating process. Using bottom spray with a Wurster insert, 50 g pellets were coated in a laboratory-scale fluid bed coater (Hanse, Changzhou, China). The process parameters were as follows: inlet temperature 50 °C, material temperature 40 °C, atomization pressure 0.15 MPa, spray rate 0.8 mL/min, and air flow rate 35 m^3^/h.

#### 2.5.2. Preparation of the Diffusion-Rate Controlling Layer

ADEC was separately used as the diffusion-rate controlling layer. Mainly, aqueous dispersion of Surelease^®^ was diluted in purified water to achieve a solid content of 15% (*w/w*), and was stirred for 45 min before coating. Then the aqueous dispersion was sprayed on pellets sub-coated with the dissolution-rate controlling layer in the same equipment. The process parameters for ADEC coating were as follows: inlet temperature 54 °C, material temperature 42 °C, atomization pressure 0.18 MPa, spray rate 1.0 mL/min and air flow rate 40 m^3^/h. After the coating process, pellets were cured in a hot oven at 60 °C for 16 h.

### 2.6. Experimental Design

A three-factor, three-level Box-Behnken experiment design was applied to evaluate the effects of different parameters on drug release rate. Briefly, the design is equal to the three replicated centre points and the set of points lying at the midpoint of each surface of the three-dimensional cube that defines the region of interest of each parameter. The three independent variables (*X*_1_, *X*_2_ or *X*_3_) were the concentration of CA in the sub-coating aqueous dispersion (*X*_1_), the sub-coating weight gain based on the dry uncoated pellet mass (*X*_2_), and the ADEC coating weight gain based on the sub coated pellets mass (*X*_3_). Each variable was coded to be in the range of −1, 0, 1, which represented different variable levels. Levels of the factors and constraints for the in vitro drug release based on preliminary pharmacokinetic study are listed in Table 1. The design required 15 experimental formulations. The independent variables of each formulation and their responses are listed in Table 2. The response surface model generated by the design is given as Equation (1):

*Y = a*_0_* + a*_1×1_* + a*_2_*X*_2_* + a*_3_*X*_3_* + a*_4_*X*_1_*X*_2_* + a*_5_*X*_2_*X*_3_* + a*_6_*X*_1_*X*_3_* + a*_7_*X*_1_^2^* + a*_8_*X*_2_^2^* + a*_9_*X*_3_^2^** (1)

where *Y* is the response parameter, *X*_1_, *X*_2_, and *X*_3_ are the independent parameters, *a*_0_ is the intercept, *a*_1_ − *a*_3_ are the main effect coefficients, *a*_4_ − *a*_9_ are coefficients of parameters with interaction or quadratic effects. Statistical analysis of the model was performed in Design-Expert software (V.8.0.6, Stat-Ease Inc., Minneapolis, MN, USA). The regression models of *Y*_1_, *Y*_2_, and *Y*_3_ were evaluated in terms of statistically significant coefficients using analysis of variance (ANOVA) and *r*^2^ values. Only coefficients with *p* values less than 0.05 were constructed in the models. In addition, response surface plots were performed to visualize the effect of parameters and their interactions on the responses. Design space, which was determined from the common region of successful operating ranges for the responses, was established following the obtained response surface to clarify the optimal formulation.

### 2.7. In Vitro Release of LXP and CA 

A dissolution test was carried out at 37 °C in 900 mL water, using a dissolution apparatus (78X-6A, Huanghai medicine inspecting institute, China) with the basket rotation speed of 100 rpm, which is specified in China Pharmacopoeia. The prepared sustained-release pellets containing 90 mg of anhydrous LXP were added to the dissolution apparatus. At pre-determined intervals, 5 mL of the sample was withdrawn and replaced with fresh medium. Then the samples were analyzed by HPLC.

In order to better understand the impact of CA on the drug release rate, simultaneous release profiles of CA and LXP in formulations with different CA concentrations were conducted. Additionally, the impact of dissolution media on the release of CA and LXP were investigated by performing the dissolution tests in the following media: pH 1.0 HCl, pH 4.5 and 6.8 phosphate buffers, and water. The contents of CA and LXP in the formulations were determined by HPLC.

### 2.8. Release Mechanism Studies

The in vitro release mechanisms of LXP were analyzed by seven kinetics models. As shown in Table 3, *Q_t_* is the release amount of LXP at time *t*, *Q*_0_** is the initial amount of LXP in the pellets, *k*_0_** is the zero order release constant and *k*_1_** is the first order release constant, *k_H_* is the Higuchi dissolution constant, *n* is exponent constant characterizing different release mechanisms, *a* is a time scale parameter and *b* is a shape parameter that characterizes the curves of the release profiles. The dissolution data of LXP were fitted to these models by linear or non-linear least-squares fitting methods. The correlation coefficients calculated by regression analysis were used to evaluate the goodness of fit for each model. 

### 2.9. Morphology Study

Scanning electron microscopy (S-8000; Hitachi High-Technologies Europe, Krefeld, Germany) was used to evaluate the morphology of the surface and cross-section of coating pellets. Samples were fitted on the copper sample holder with a double sided adhesive tape, sputter coated with a 10-nm thick gold layer under argon atmosphere.

### 2.10. The Pharmacokinetic Studies

#### 2.10.1. Administration Programme

All animal treatments were performed in accordance with the Regulations of the Administration of Affairs Concerning Experimental Animals and the study protocol was admitted by the Ethics Committee of China Pharmaceutical University (Approval No. 2018-0315). An open label, randomized, two-period crossover experiment design with one week wash-out period was used in this study. Six male beagle dogs (weight 8.7 ± 1.1 kg), fasted but free access to water for 12 h prior to the experiment, were used in the study. Pellets of the optimal formulation were filled into hard gelatin capsules. The immediate release tablet (Loxonin^®^, 60 mg anhydrous LXP) and the capsule of the optimal formulation (90 mg anhydrous LXP) were administered to beagles in the morning with 100 mL water. Then, 6 h after dosing, dogs were provided with standard food.

A total of 2 mL of the blood samples were withdrawn before and then 0.5, 1.0, 2.0, 3.0, 4.0, 5.0, 6.0, 8.0, 10.0, and 12.0 h after dosing via cannulated needle from front legs. Plasma was obtained by centrifuging the blood at 4000 rpm for 15 min, and was kept frozen at −20 °C before analysis. 

#### 2.10.2. Determination of LXP in Plasma

A stable and selective HPLC method, modified by previous papers [12,35], was applied for the analysis of LXP in dog plasma. Pretreatment was carried out by adding 50 μL of internal standard (100 μg/mL ketoprofen in acetonitrile), 50 µL of zinc sulfate solution (10%, *w/w*) and 750 μL acetonitrile into 500 μL plasma sample. After vortex-mixing for 1 min, sample was centrifuged at 8000 rmp for 15 min. Then 10 μL of the supernatant was injected into the HPLC system. The separation was performed on an Inertsil C18-ODS column (5 µm, 4.6 × 150 mm, Shimadzu, Japan) with a guard column (4.6 × 10 mm, 5 μm particle size, ANPEL Laboratory Technologies Inc, Shanghai, China) at a flow rate of 1 mL/min. Additionally, the mobile phase was a mixture of acetonitrile and 0.05 M monopotassium phosphate (35:65, *v/v*), adjusted to pH 3.0 with phosphoric acid. Chromatograms were recorded at 223 nm with a Shimadzu-SPD detector. The linear range of this method was 0.1–20.0 µg/mL with an *r*^2^ value of not less than 0.999. The lower limit of quantification (LLOQ) was 100 ng/mL and the extraction recoveries of high, middle, and low concentrations of LXP were 102.5 ± 2.0%, 97.1 ± 2.5% and 97.1 ± 5.9%, respectively. The R.S.D.s reflecting the intra-day and inter-day precision of LXP were less than 11.77%.

#### 2.10.3. Bioavailability Study

Non-compartmental pharmacokinetic analysis was applied to calculate parameters such as T_max_, C_max_, AUC_0–t_, and AUC_0–∞_ from the plasma concentration-time curve data using WinNonlin software (version 1.5, Pharsight Corp. Mountain View, CA, USA). The relative bioavailability of the optimal formulation to the commercial tablet (reference) was calculated using the following Equation (2):

(2) $$\mathrm{Relative}.\mathrm{bioavaibility}=\frac{AUC_{T}{}_{0-\infty }\times X_{R}}{AUC_{R0-\infty }\times X_{T}}\times 100\%$$

where *X_R_* and *X_T_* were the administered dose of the reference and test respectively. Results were presented as means ± standard deviation. A one-way ANOVA (SPSS, version 19) with *p* < 0.05 as a level of significance was applied to examine the differences of C_max_ and AUC_0–∞_ between the test and reference. 

## 3. Results and Discussions

### 3.1. Impact of CA on Drug Release

**Figure 2:**
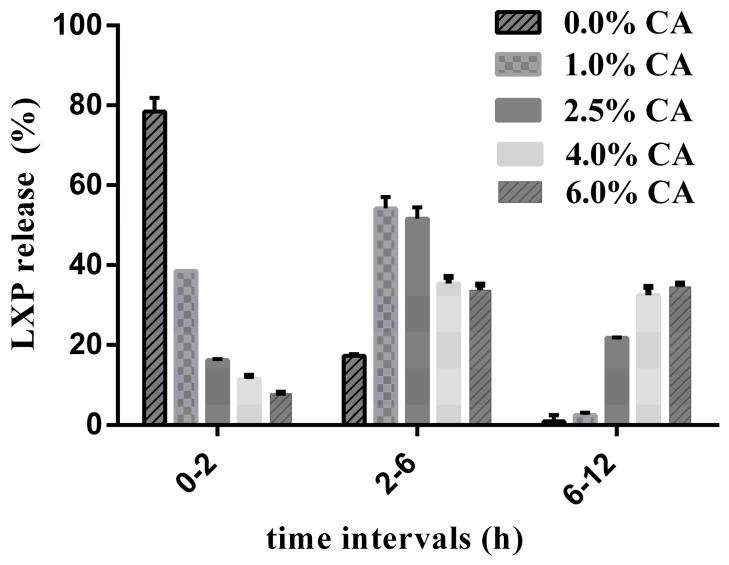
Effect of citric acid concentration on the drug release within different intervals.

Formulations with different concentrations of CA in the sub-layer were developed to evaluate the effect of pH-modifier on the drug release rate, while the dissolution-controlling layer and ADEC coating levels were kept at 8% and 11% respectively. The results in Figure 2 illustrated that formulation without CA showed a fast release of LXP (>80% within 2 h), while the drug release within 2 h was decreased to 40% at a CA concentration of 1%. Additionally, the release rate continued to decrease with the increase of CA concentration, which showed 16.37%, 11.34%, and 7.77% of LXP release within the first 2 h. At the CA concentration of 1%, a completed drug release was finished within 6 h. While at higher CA concentrations, there were still 21.80% (2.5% CA) and 32.43% (4.0% CA) of the initial drug amount released after 6 h. 

As a pH modifier, CA was aimed to modulate the pH_M_ inside the systems. For pH-sensitive compound, its solubility is more appropriate to be described as the solubility in the diffusion layer at the surface of the dissolving particles [25]. Therefore, according to the Noyes–Whitney theory, the dissolution rate of LXP was much more dependent on the solubility in the low pH_M_ beneath the diffusion-controlling layer, other than the dissolution media. Theoretically, drug release rate from a coherent film coating system is controlled by both the coating level and the drug concentration gradient across the coating film, which obeyed the Fick’s diffusion law. As the film coating level was kept constant, drug release rate was predominantly controlled by the drug concentration gradient, which was determined by the dissolution rate of LXP inside the pellets. Therefore, as the drug release was significantly decreased with the increase of CA concentrations (Figure 2), the first step of developing a dissolution-rate controlling layer proved to work.

**Figure 3:**
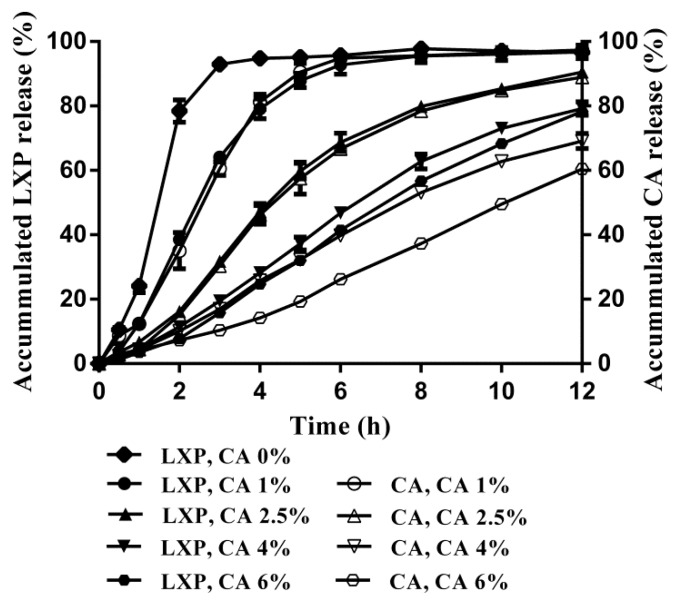
The simultaneous release profiles of citric acid and loxoprofen from sustained release pellets at different concentrations of citric acid (*n* = 3).

Furthermore, simultaneous release profiles in Figure 3 were constructed to investigate the impact of dynamic release process of CA on the drug release rate. In formulations with lower CA concentrations, with the release of CA during the dissolution period, pH_M_ could be changed from 0.4 (the saturated solution pH of CA) to the approximate equilibrium pH of the dissolution medium [36]. As the solubility of LXP changed approximately 300 times within this pH range (Supplementary Materials Figure S1), the drug dissolution rate was significantly dependent on the amount of CA left inside the pellets. Therefore, matching release profiles of LXP and CA were observed at low concentrations of CA. 

While in formulations with higher CA concentrations, as a sufficient amount of CA remained inside the pellets at the end of the dissolution period, e.g., 30% or 40% of the initial CA amount left at the concentration of 4% or 6% respectively, a constant and effective pH_M_ was achieved inside the pellets, which resulted in no further decrease of the drug release rate (Figure 3). A similar phenomenon was observed in a matrix tablet containing dipyridamole, due to a constant and effective pH_M_ maintained by fumaric acid inside the dosage form, no further enhancement of the dipyridamole release was observed after increasing the fumaric acid concentration to 40% [37]. Therefore, a discrepancy of the release profiles between CA and LXP was observed in formulations with higher CA concentrations.

### 3.2. Release Experiments and Statistical Evaluation

#### 3.2.1. Testing of Drug Release

**Figure 4:**
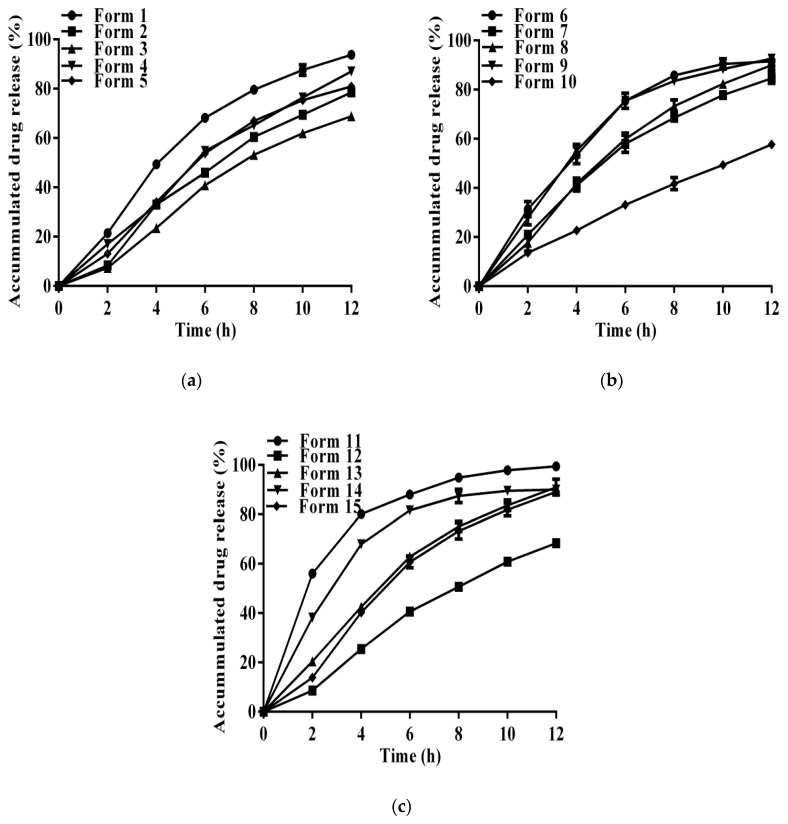
Dissolution profiles of loxoprofen in formulations prepared by the Box-Behnken design experiments (**a**) Formulation Nos. 1–5, (**b**) Formulation Nos. 6–10, (**c**) Formulation Nos. 11–15.

Experimental variables and observed responses of all the 15 formulations were listed in Table 2. And their drug dissolution profiles were displayed in Figure 4. At a low level of CA concentration, most of the formulations (Formulation Nos. 6, 9, 11) showed a fast drug release except Formulation No. 7, which had a high coating level of ADEC. The fast release in Formulation Nos. 6, 9, 11 was attributed to an inefficient pH_M_ inside the pellets and the short diffusion pathway of ADEC coating, which could be identified as the failure of the first- and second-step control. As a prolonged diffusion pathway was developed in Formulation No. 7, the release rate of LXP was significantly decreased. While at a low coating level of ADEC, most of the formulations (Formulation Nos. 1, 11, 14) showed a fast drug release rate, expect Formulation No. 13, which was incorporated with a high concentration of CA. The fast release in Formulation Nos. 1, 11, 14 could be explained by a failure of the second-step control, as a short diffusion pathway created by the low coating level of ADEC was unable to retard the release rate of LXP. However, when 3% of the CA concentration was applied in Formulation No. 13, the effective control of the drug dissolution-rate could compensate for the failure of the diffusion-rate control to achieve a sustained release of LXP.

#### 3.2.2. Regression Equations

Based on the experiment data, the coefficients and their *p*-values of the fitted full quadratic equations calculated by Expert-Design 8.0.6 (Stat-Ease Inc., Minneapolis, MN, USA) are listed in Table 4. The final equations consisted of only statistically significant coefficients. It is clear that the citric acid concentration (*X*_1_) and ADEC coating weight gain (*X*_3_) showed significant effects on the drug release rate throughout the dissolution period, while the weight gain of the dissolution-rate controlling layer (*X*_2_) showed only a weak effect during the dissolution period. The impact of *X*_1_ on the drug release rate verified the effectiveness of the first-step control on the drug release rate, which could be explained by its impact on the pH_M_, as extensively reported in the literature [36,37]. The effect of *X*_3_ on the drug release rate could be attributed to its control on the drug diffusion rate, which was considered as the second-step control [38]. Besides, an interaction effect of *X*_1_ and *X*_3_ was observed on the response of *Y*_1_ and *Y*_3_, which might be explained by a speculation that *X*_3_ also showed an effect on the release rate of citric acid. Additionally, it seemed that *X*_3_ played a more dominant role on the final drug release, as high coefficients of the main, interaction, and quadratic effects of *X*_3_ were observed in *Y*_3_. From the statistic results in Table 4, we could conclude that both the dissolution-rate and diffusion-rate controlling steps have significant effects on the drug release rate.

#### 3.2.3. Response Surface Plots

**Figure 5:**
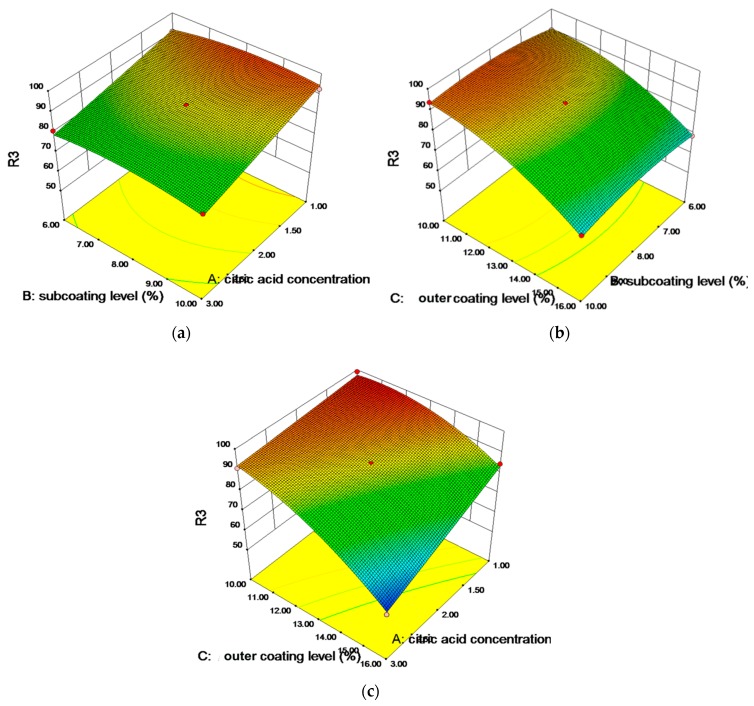
Contour plots showing the effects of (**a**) *X*_1_ and *X*_2_, (**b**) *X*_2_ and *X*_3_, and (**c**) *X*_1_ and *X*_3_ on the response *Y*_3_.

The relationship between the dependent and independent variables was further elucidated using a 3D response surface plot, which is useful to see the effect of two factors on the response at one time while the third factor is kept at a constant level. The effects and interactions between concentration of citric acid (*X*_1_), the sub coating weight gain (*X*_2_), and ADEC coating weight gain (*X*_3_) on the finial drug release (*Y*_3_) are given in Figure 5. The similar impacts of the three factors on the other responses (*Y*_1_ and *Y*_2_) can be seen in Supplementary Materials Figures S2 and S3. As illustrated in Figure 5a,b, it was clear to see that *X*_2_ showed little effect on *Y*_3_ irrespective of the levels of other two factors. This was attributed to the fact that the dissolution-rate controlling layer is made up of aqueous polymer, which was dissolved before 12 h. 

The effects of citric acid concentration (*X*_1_) and the ADEC coating weight gain (*X*_3_) on *Y*_3_ are depicted in Figure 5c. While *X*_3_ was kept at low level, the increase of *X*_1_ from 1% to 3% showed no effect on *Y*_3_, which kept nearly constant at above 90%. While at a high level of *X*_3_, the increase of *X*_1_ from 1% to 3% resulted in a significant decrease of *Y*_3_ from 85% to 60%. The result indicated that an interaction effect of the two factors existed on the drug release rate, as mentioned in Section 3.2.2. At a high level of *X*_3_, an effective diffusion barrier was formed on the surface of the pellets [38], which significantly reduced the diffusion rate of loxoprofen. However, the release rate of loxoprofen was not solely controlled by the diffusion-controlling layer. For example, a nearly complete release of loxoprofen (>85%) was observed at a high level of *X*_3_ (Figure 5c), when *X*_1_ was kept at a low level of 1%. Therefore, the release rate of loxoprofen was a combined result of the two controlling steps. At a low level of *X*_3_, the citric acid was soon released regardless of its concentrations, which resulted in a quick increase of the pH_M_ and a fast drug release rate. When the concentration of citric acid and ADEC coating weight gain were kept at high levels, both the drug dissolution and diffusion rate were reduced, which resulted in a prominent decrease of the drug release rate.

#### 3.2.4. Design Space and Formulation Parameters Optimization

**Figure 6:**
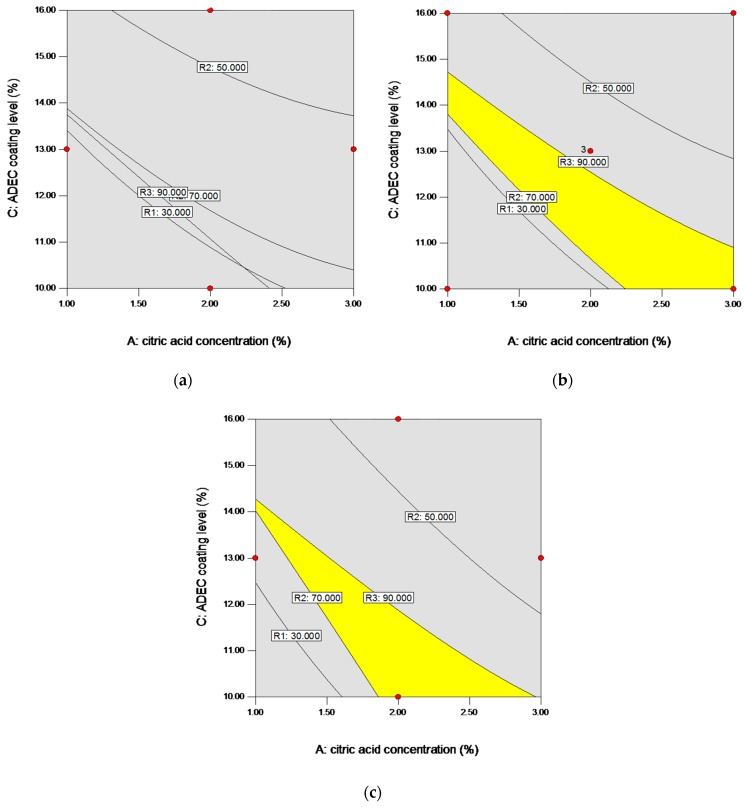
Design space of operating variables of the citric acid (CA) concentration and aqueous dispersion of ethyl cellulose (ADEC) coating level (**a**) at the low level of the sub-layer coating weight gain, (**b**) at the medium level of the sub-layer coating weight gain, and (**c**) at the high level of the sub-layer coating weight gain (yellow zone: design space; grey zone: failure space).

Design space was defined by the ICH Q8 as the relationship between the process inputs (material attributes and process parameters) and the critical quality attributes that have been demonstrated to provide assurance of quality [39]. The wider the design space is, the more robust and flexible the process is to resist variations [40]. As the response surface models of the output parameters as a function of selected variables were given, design space of *X*_1_, *X*_2_, and *X*_3_ was determined by applying constraints on *Y*_1_ (<30%), *Y*_2_ (50–70%), and *Y*_3_ (>90%). The yellow overlap region of ranges for the three responses in Figure 6a–c show the proposed design space of the citric acid concentration *X*_1_ and the ADEC coating weight gain *X*_3_ at three different levels of the sub-coating weight gain *X*_2_. As shown in Figure 6a, there was no design space of *X*_1_ and *X*_3_ at the low level of *X*_2_. Additionally, Figure 6c depicted a narrow design space of *X*_1_ and *X*_3_ at high level of X_2_, which would increase the difficulty of the operation process since an accurate coating load of ADEC must be achieved during the manufacturing process. While at the medium level of *X*_2_ (Figure 6b), the design space was expanded, which showed a less strict field of ADEC coating level. As the design space depicted the ranges of the formulation parameters for achieving the desired quality of product, the levels of the three factors for the optimal formulation must be set within the design space. Considering the robustness and flexibility, parameters of the optimal formulation were set at the medium level of sub coating weight gain with the CA concentration and coating level of ADEC at 2.5% and 11.0% respectively. The model predicted a release profile of 19.87% at 2 h, 64.48% at 6 h, and 91.71% at 12 h. To verify these values, a new batch of the optimal formulation was prepared. The obtained release data of the optimal formulation were in close agreement with the predicted values with a maximum percentage error of 11.73% at the initial release (data not showed).

### 3.3. Simultaneous Release of CA and LXP from the Optimal Formulation in Different Dissolution Media

**Figure 7:**
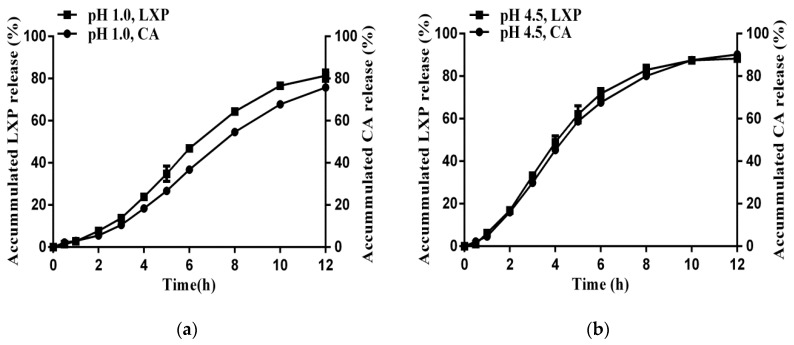

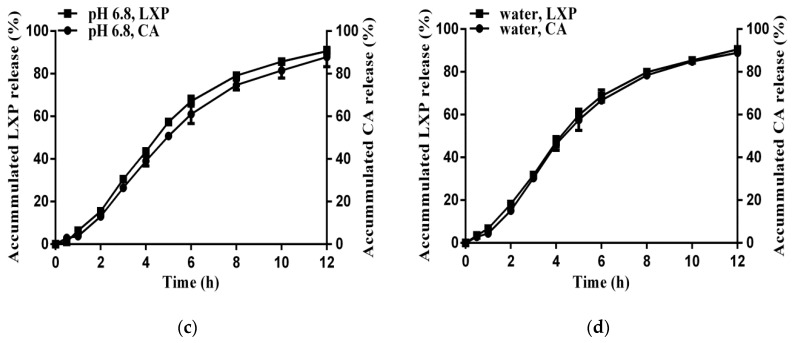
Loxoprofen and citric acid released from sustained release pellets in different dissolution media. (**a**) pH 1.0 HCl (**b**) pH 4.5 phosphate buffer (**c**) pH 6.8 phosphate buffer (**d**) water (means ± SD, *n* = 3).

In order to evaluate the effect of pH on drug release, various media simulating different physiology pH values were applied. As shown in Figure 7, dissolution tests were performed in pH 1.0 HCl, pH 4.5 and pH 6.8 phosphate buffer solutions and water. Furthermore, the release profiles of CA were also investigated in these media. As illustrated in Figure 7, drug release profiles were pH-independent at pH above 4.5, and showed similar release profiles to that of CA. 

Although the solubility of LXP was pH-independent in media with pH above 4.5 (Supplementary Materials Figure S1), it seemed that the dissolution media were not the reason for this pH-independent release behavior. As the drug showed a completed release within 3 h without the incorporation of CA inside the pellets (Figure 3, formulation with the CA concentration of 0%), it should exhibit a similar release for the optimal formulation as the dissolution media were also above 4.5. In the contrast, the optimal formulation showed sustained release for almost 12 h. It was the pH_M_ created by CA, which showed similar release profiles at pH above 4.5, that accounted for the pH-independent release profiles of LXP (Figure 7). As mentioned before, the saturated solution pH of CA was 0.4 [36], which was much lower than that of the dissolution media except the pH 1.0 HCl. However, with the release of CA during the dissolution period, the amount of CA left inside the optimal pellets was insufficient to maintain a constant pH_M_ inside the pellets. The pH_M_ was gradually increased, which resulted in a consistent enhancement of the drug solubility. In addition, matching sustained release profiles of LXP and CA were achieved in Figure 7. 

While at pH 1.0, it was the dissolution media that showed a major effect on the drug release rate. With the decrease of CA, the pH_M_ would soon be increased up to above 1.0. However, the dissolution media that penetrated into the pellets provided a stable pH_M_ inside the pellets, which in turn resulted in a different release profile to the other three. In conclusion, the dissolution media and the incorporated CA played a combined effect on the drug release rate. In media with high pH values, the CA showed a greater effect on the drug release rate. While in media with low pH values, it was the dissolution media that dominated the drug release rate.

### 3.4. Release Mechanism Studies

In order to elucidate the transport mechanism of LXP in the optimal formulation, different mathematical models were applied to analyze the kinetics of the release data. As shown in Table 5, the incorporation of CA into the sub-layer resulted in abnormal release kinetics of this ADEC coating system, as the *n* value for Ritger–Peppas was 0.7422, which is between 0.45 and 0.89, indicating a non-Fick diffusion [41]. Additionally, a general empirical equation of Weibull distribution model with *r^2^* of 0.9944 was more appropriate to describe the release process of the optimal formulation. In the model, the derived estimate of *b* value was calculated to be 1.38, which represented a sigmoid shape curve (*b* > 1) for the release profile [41]. The initial slow release representing the starting part of the sigmoid curve was a result of several factors. As reported previously, the permeability of water through the EC coating is much faster than the permeability of the compound [38], which might contribute to the initial slow release of LXP. Besides, the decreased dissolution rate created by the initial low pH_M_ inside the pellets also played an important role on the initial slow release. Furthermore, the hydration of the hydroxypropyl methyl cellulose (HPMC) inside the pellets during the initial dissolution period could also inhibit the initial drug release rate. Thereafter, due to the saturation of water inside the pellets and the disruption of the dissolution-rate controlling layer, the drug release rate was dominated by the dissolution- and diffusion-rate control, which resulted in a sustained release profile. 

### 3.5. Scanning Electron Photomicrographs

**Figure 8:**
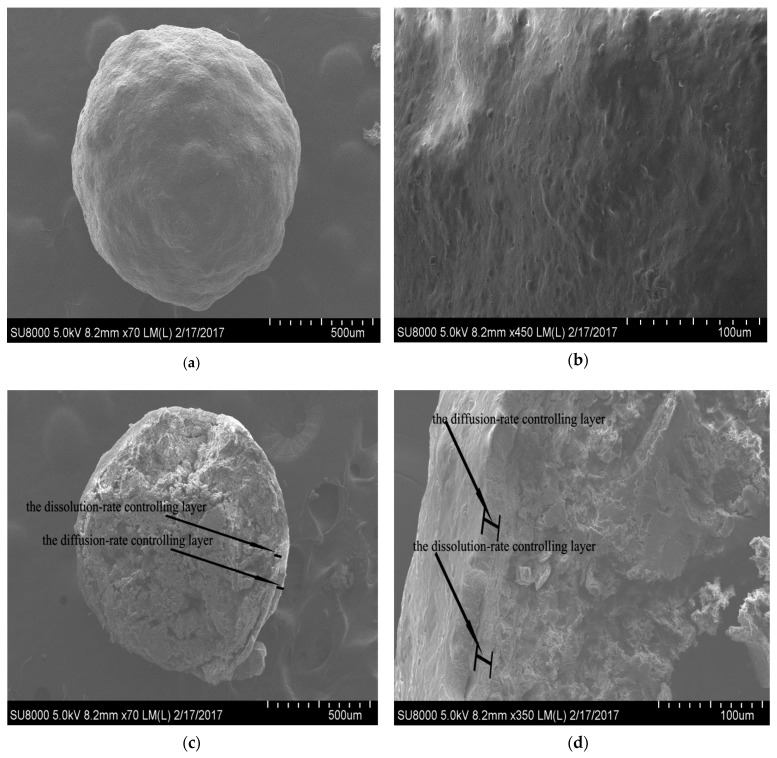
SEM photographs of pellets with double coating layers: (**a**) Surface of sustained release pellets with 70 magnifications, (**b**) surface of sustained release pellets with 450 magnifications, (**c**) cross-section of sustained release pellets with 70 magnifications, (**d**) cross-section of sustained release pellets with 350 magnifications.

Figure 8 shows the scanning electron photomicrographs of the optimal pellets. The surface of pellets were smooth (Figure 8a,b), and no crack could be seen. Besides, layers of the dissolution-rate controlling layer and the diffusion-rate controlling layer were clearly seen in the cross-section of the coating pellets (Figure 8c,d). These results indicated that a successful procedure had been developed for manufacturing the sustained release pellets. 

### 3.6. Pharmacokinetic Studies

**Figure 9:**
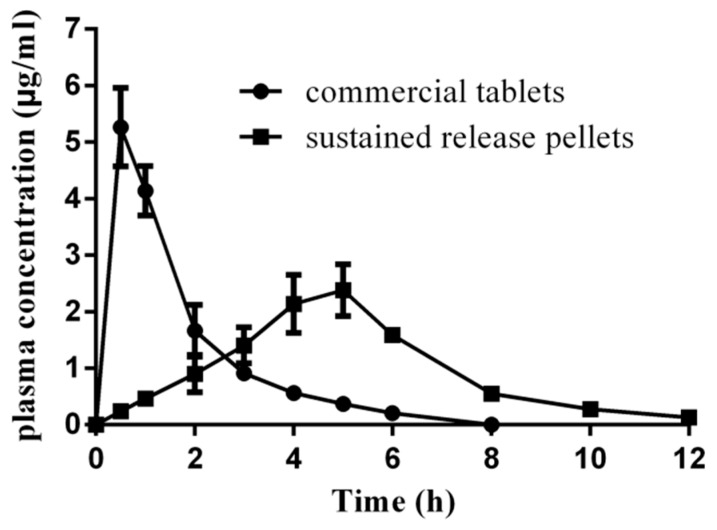
Plasma drug concentrations vs. time after oral administration of conventional tablets (60 mg) and sustained release pellets (90 mg).

The pharmacokinetic studies of the optimal pellets and the commercial tablets were investigated on fasted beagles. The profile of mean plasma concentrations of LXP versus time is shown in Figure 9. The main pharmacokinetic parameters are summarized in Table 6. As shown in Figure 9, the plasma concentration of the commercial tablet quickly increased and reached the peak concentration of 5.16 μg/mL at 0.5 h after administration. Then it dropped down and was only 0.2 µg/mL at 6 h. This was attributed to the short half-life (t_1/2_ = 64.46 min) of LXP [12], which resulted in a quick elimination of the drug in vivo. The optimal formulation reached the maximum plasma concentration of 2.40 µg/mL at 5 h after administration, and the drug concentration fell slowly even at 12 h, when the drug concentration was 0.15 µg/mL, in contrast with the undetectable drug concentration in plasma for the commercial tablet 8 h after administration. 

As a pro-drug, LXP inhibits the activities of cyclooxygenase-1 and -2 (COX-1 and COX-2) by its active metabolite trans-OH LXP, of which the IC_50_ values for COX-1 and COX-2 were 0.38 and 0.12 µM, respectively [42]. It has been reported that the concentration of trans-OH LXP, which was the major metabolite of LXP, was equal to more than half of the LXP concentration detected in plasma [12]. As the LXP concentration range in plasma of the optimal pellets was 0.6–10.0 µM, we can deduce that the concentration of trans-alcohol LXP after administration of the optimal pellet would be higher than the IC_50_ of the trans-OH LXP. Therefore, a therapy concentration of trans-OH LXP in plasma after administration of the optimal pellets would be maintained for almost 12 h. As the frequency of dosage of the optimal formulation was reduced to two times a day, the patient’s compliance would be better improved. It has been reported that the incidence of gastric lesions after administration of LXP in rats showed a dose-dependent manner. Additionally, the amount of PGE_2_, which has a strong protective effect on the GI mucosa, also decreased in a concentration-dependent manner after treatment of LXP within the concentration range of 1.0 µM to 1.0 M [14,43]. Therefore, the risk of GI lesions would be significantly decreased, since the initial burst release disappeared in vivo and the C_max_ of LXP was significantly decreased from 20 to 10 µM after administration of the optimal formulation. Besides, a less fluctuant drug concentration in plasma was achieved for the optimal pellets in Figure 9. The significant difference (*p* < 0.05) of AUC_0–∞_/dose between the test formulation and conventional tablet might be caused by the limited GI absorption site of LXP, which would need further investigation. The relative bioavailability of the test formulation was 87.16% compared with the reference, and it would be improved in patients as a prolonged GI transit time has been reported in humans [44].

## 4. Conclusions

In conclusion, this article provided a novel concept of two-step control of the release rate of pH-sensitive drugs. Additionally, the results of the drug in vitro release profiles proved that both the dissolution-rate controlling step created by the sub-layer containing CA and the diffusion-rate controlling step developed by the ADEC coating showed significant effects on the release rate of LXP. In addition, the amount of the acid modifier in the optimal formulation, which accounted for only approximately 3% of the total preparation weight, was dramatically decreased compared with other formulations containing acidic modifiers. The in vivo studies revealed that this novel two-step control system could achieve a more stable and sustained release plasma concentration of LXP compared with the immediate release tablet.

## Figures and Tables

**Table 1:** The factors and responses of the Box-Behnken design.

Independent Variables	Levels Used		
	−1	0	1
*X*_1_ = citric acid concentration (%)	1	2	3
*X*_2_ = subcoating weight (%)	6	8	10
*X*_3_ = ADEC coating weight (%)	10	13	16
Responses	Constraints		
*Y*_1_ = the drug release within 2 h	<30%		
*Y*_2_ = the drug release within 6 h	50–70%		
*Y*_3_ = the drug release within 12 h	>90%		

**Table 2:** Independent variables and observed responses of the Box-Behnken design.

Formulations	Factors (%)			Responses (%)		
	*X* **_1_**	*X* **_2_**	*X* **_3_**	*Y* **_1_**	*Y* **_2_**	*Y* **_3_**
1	2.0	10.0	10.0	21.5	71.2	93.8
2	3.0	10.0	13.0	8.4	46.0	78.5
3	2.0	6.0	16.0	7.4	40.9	68.9
4	2.0	8.0	13.0	17.1	54.8	87.0
5	3.0	6.0	13.0	13.1	53.8	81.0
6	1.0	6.0	13.0	31.4	75.0	91.0
7	1.0	8.0	16.0	21.0	58.0	86.0
8	2.0	8.0	13.0	17.7	59.7	89.0
9	1.0	10.0	13.0	27.8	75.5	92.6
10	3.0	8.0	16.0	13.6	33.1	57.7
11	1.0	8.0	10.0	56.0	88.0	99.0
12	2.0	10.0	16.0	8.6	40.2	68.3
13	3.0	8.0	10.0	20.5	60.2	91.0
14	2.0	6.0	10.0	38.2	81.6	90.0
15	2.0	8.0	13.0	13.9	60.7	89.0

**Table 3:** Models for drug release.

Model Name	Equation
Zero-order model	*Q_t_ = k_0_t*
First-order model	*ln(Q_0_ − Q_t_) = −k_1_t + Q_0_*
Higuchi diffusion model	*Q_t_ = k_H_t^1/2^*
Ritger–Peppas model	*lnQ_t_ = n lnt + k*
Weibull distribution model	*log[−ln(1 − Q_t_)] = b logt − loga*
Hixson–Crowell model	*(1 − Q_t_)^1/3^ = 1 − kt*
Baker–Lonsdale model	*3/2 [ 1 − (1 − Q_t_)^2/3^] − Q_t_ = kt*

**Table 4:** Regression coefficients and associated *p*-values of the fitted models.

Term	Drug Release Within 2 h (*Y*_1_)		Drug Release Within 6 h (*Y*_2_)		Drug Release Within 12 h (*Y*_3_)	
	Cofficient	*p*-Value	Cofficient	*p*-Value	Cofficient	*p*-Value
Constant	16.23	0.000	58.40	0.000	88.33	0.000 *
*X*_1_	−10.08	0.001 *	−12.91	0.001 *	−7.38	0.000 *
*X*_2_	−2.98	0.007 *	−2.30	0.091	0.29	0.697
*X*_3_	−10.70	0.001 *	−16.11	0.001 *	−11.79	0.000 *
X_1_*X*_2_*	−0.27	0.787	−2.07	0.240	−1.02	0.347
X_2_*X*_3_*	4.47	0.006 *	2.42	0.180	−1.10	0.316
X_1_*X*_3_*	7.03	0.000 *	0.70	0.672	−4.72	0.005 *
X_1_*X*_1_*	6.40	0.001 *	2.78	0.148	0.13	0.902
X_2_*X*_2_*	−2.45	0.058	1.40	0.427	2.69	0.047 *
X_3_*X*_3_*	5.15	0.004 *	−1.33	0.451	5.39	0.003 *
Regression equation	*Y*_1_ = 16.23 − 10.08*X*_1_ − 2.98*X*_2_ − 10.7*X*_3_ − 4.47*X*_2_*X*_3_** + 7.03*X*_1_X_3_** + 6.4*X*_1_^2^** + 5.15*X*_3_^2^**		*Y*_2_ = 58.40 − 12.91*X*_1_ − 16.11*X*_3_		*Y*_3_ = 88.33 − 7.38*X_1_* − 11.79*X*_3_ − 4.72*X*_1_*X*_3_** − 2.69*X*_2_^2^** − 5.39*X*_3_^2^**	
R-Squared	0.9921		0.9865		0.9891	

^*^p-value < 0.05.

**Table 5:** Models simulated for the drug release profiles of the optimal formulation.

Content	Model	Equation	*r^2^*
loxoprofen	Zero-order model	*Q_t_ = 0.0833t + 0.0238*	0.9288
	First-order model	*ln(Q_0_ − Q_t_) − lnQ_0_ = −0.1787t + 0.0814*	0.9794
	Higuchi diffusion model	*Q_t_ = 0.3126t^1/2^ − 0.1496*	0.9454
	Ritger–Peppas model	*lnQ_t_ = 0.7422 lnt + 2.7562*	0.9597
	Weibull distribution model	*log[−ln(1 − Q_t_)] = 1.3840 logt − 1.0449*	0.9944
	Hixson–Crowell model	*(1 − Q_t_)^1/3^ = −0.0514t + 1.0167*	0.9874

**Table 6:** The pharmacokinetic parameters of loxoprofen after oral administration of the optimal sustained release pellets and commercial tablets in beagle dogs (*n* = 6).

Pharmacokinetic Parameters	*C_max_* (µg/mL)	*T_max_* (h)	*AUC*_0–12_ (µg h/mL)	*AUC*_0–∞_ (µg h/mL)	Relative Bioavailability (%)
Optimal pellets (90 mg)	2.60 ± 0.23	4.80 ± 0.57	12.77 ± 0.88	13.48 ± 0.94	87.16 ± 0.07
Commercial tablets (60 mg)	5.16 ± 0.60	0.60 ± 0.22	9.73 ± 0.61	10.31 ± 0.45	-

## Supplementary Materials

The following are available online at https://www.mdpi.com/1999-4923/11/6/260/s1, Figure S1: pH solubility profiles of loxoprofen at 25 °C. Figure S2: Contour plots showing the effects of (**A**) *X*_1_ and *X*_2_, (**B**) *X*_2_ and *X*_3_, and (**C**) *X*_1_ and *X*_3_ on the response *Y_1_*. Figure S3: Contour plots showing the effects of (**A**) *X*_1_ and *X*_2_, (**B**) *X*_2_ and *X*_3_, and (**C**) *X*_1_ and *X*_3_ on the response *Y*_2_.
