# Supplementary material for: Development and In Vitro-In Vivo Evaluation of a Novel Sustained-Release Loxoprofen Pellet with Double Coating Layer
Source: Pharmaceutics. 2019 Jun 5;11(6):260. doi: 10.3390/pharmaceutics11060260 (PMC6631012; doi:10.3390/pharmaceutics11060260)
Supplement: Supplementary file 1 [file pharmaceutics-11-00260-s001.pdf]

Supplementary Data:

Development and in vitro-in vivo evaluation of a novel sustained-release loxoprofen pellet with double coating layer

Dongwei Wan [1], Min Zhao [1], Jingjing Zhang [1] and Libiao Luan [2,*]

1 College of pharmacy, China Pharmaceutical University, No. 639 Longmian Road, Nanjing 211100, Jiangsu Province, China; 710176323@163.com (D.W); 19850856528@163.com (M.Z); 15651915012@163.com (J.Z)
2 College of pharmacy, China Pharmaceutical University, Xuanwumen Campus, No. 24 Tongjiaxiang, Nanjing 210009, Jiangsu Province, China
* Correspondence: luanlibiao@126.com.; Tel.: +86-18851106518

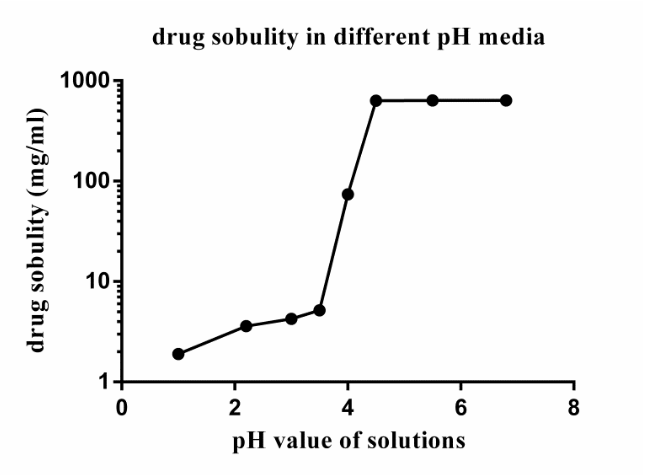

Figure. S1. pH solubility profiles of loxoprofen at 25°C.

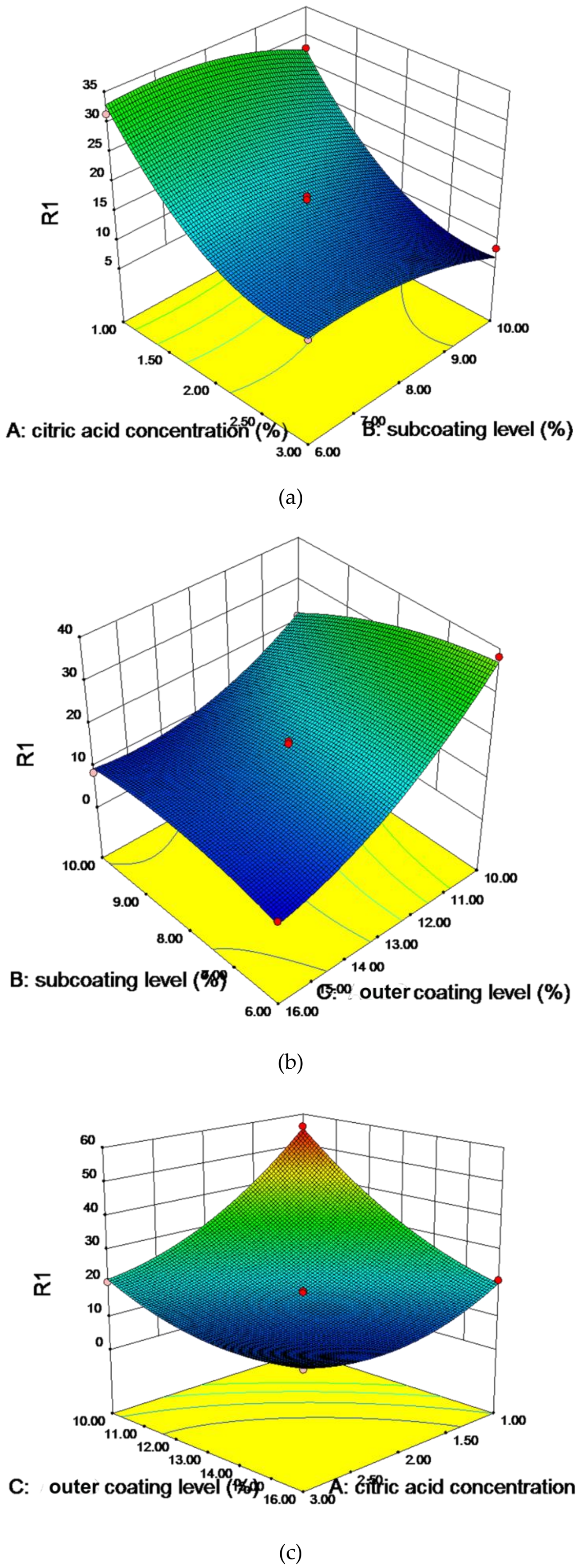

Figure. S2. Contour plots showing the effects of (**A**) X_1 and X_2, (**B**) X_2 and X_3, and (**C**) X_1 and X_3 on the response Y_1.

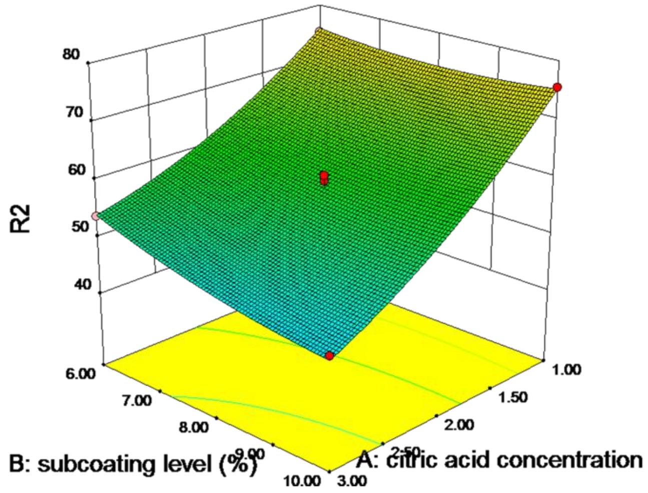

(a)

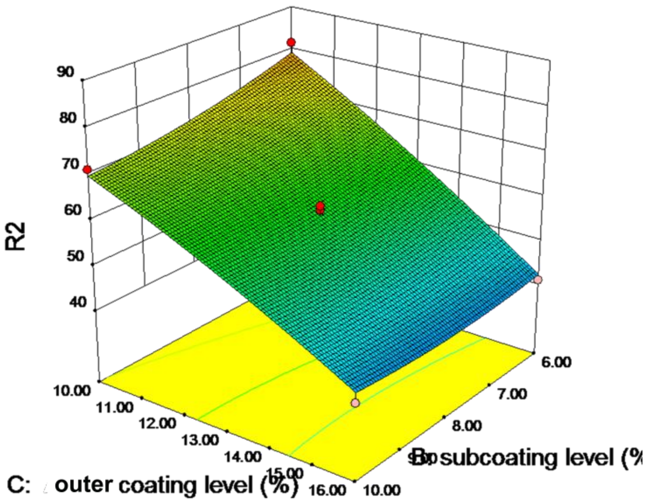

(b)

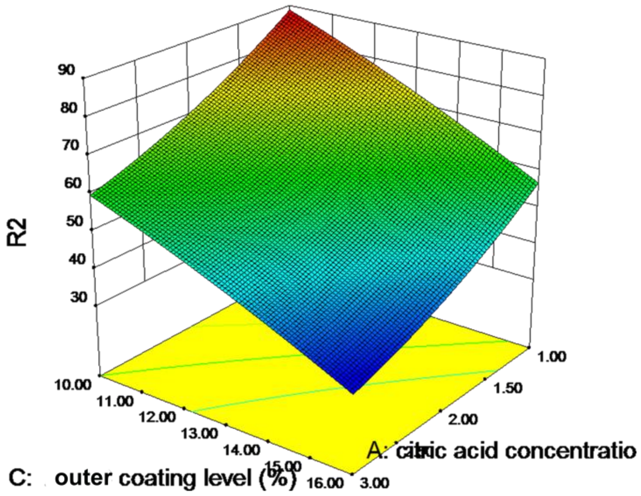

(c)

Figure. S3. Contour plots showing the effects of (**A**) X_1 and X_2, (**B**) X_2 and X_3, and (**C**) X_1 and X_3 on the response Y_2.
